# Retrospective Analysis of R-COMP Therapy in Patients with Diffuse Large B-Cell Lymphoma (DLBCL): Assessing the Impact of Sample Selection Bias

**DOI:** 10.3390/jcm14020639

**Published:** 2025-01-20

**Authors:** Chiara Romano, Francesco Branda, Nicola Petrosillo, Annalisa Arcari, Francesco Merli, Michele Spina, Giancarlo Ceccarelli, Massimo Ciccozzi, Fabio Scarpa, Luigi Rigacci

**Affiliations:** 1 Unit of Medical Statistics and Molecular Epidemiology, Università Campus Bio-Medico di Roma, 00128 Rome, Italy; chiara.romano@unicampus.it (C.R.); m.ciccozzi@unicampus.it (M.C.); 2 Infection Prevention & Control, Infectious Disease Service, Fondazione Policlinico Universitario Campus Bio-Medico, 00128 Rome, Italy; 3 Hematology Unit, Ospedale Guglielmo da Saliceto, 29122 Piacenza, Italy; a.arcari@ausl.pc.it; 4 Hematology Unit, Azienda Unità Sanitaria Locale-IRCCS di Reggio Emilia, 42123 Reggio Emilia, Italy; francesco.merli@ausl.re.it; 5 Division of Medical Oncology and Immune-Related Tumors, Centro di Riferimento Oncologico di Aviano IRCCS, 33081 Aviano, Italy; mspina@cro.it; 6 Department of Public Health and Infectious Diseases, University Hospital Policlinico Umberto I, Sapienza University of Rome, 00185 Rome, Italy; giancarlo.ceccarelli@uniroma1.it; 7 Department of Biomedical Sciences, University of Sassari, 07100 Sassari, Italy; fscarpa@uniss.it; 8 Research Unit of Hematology and Stem Cell Transplantation, Fondazione Policlinico Universitario Campus Bio-Medico di Roma, 00128 Rome, Italy; l.rigacci@policlinicocampus.it

**Keywords:** retrospective analysis, R-COMP therapy, diffuse large B-cell lymphoma, sample selection bias, cardiotoxicity risk, prognostic factors, International Prognostic Index, survival analysis, statistical methods

## Abstract

**Background**: Retrospective studies are often criticized for their susceptibility to case selection bias compared to prospective studies, which include all patients consecutively and are thus less prone to such limitations. However, the larger sample sizes typical of retrospective studies can sometimes offset this drawback. On behalf of the Fondazione Italiana Linfomi (FIL), a substantial retrospective study involving 946 patients was conducted to examine the use of non-pegylated liposomal anthracycline (Myocet). This was followed by a prospective study, the Prospective Elderly Project, which enrolled 308 patients treated with the same liposomal anthracycline regimen. **Methods:** The objective of this analysis was to determine whether the patient cohort from the retrospective study significantly differed from the cohort in the prospective study. Statistical hypothesis testing was applied to assess whether the samples from both studies originated from the same underlying population. The Anderson–Darling test, a non-parametric statistical method, was utilized to evaluate and compare the overall survival distributions between the two patient cohorts. **Results:** The statistical tests produced conflicting results, suggesting a potential selection bias in the retrospective study or the possibility that the two groups were from the same population. These discrepancies may have arisen due to the choice of statistical methods or the quality of the data analyzed. **Conclusions:** This study highlights the challenges of comparing retrospective and prospective cohorts and underscores the importance of selecting appropriate statistical methodologies. The findings provide valuable insights and lay the groundwork for developing innovative approaches to improve such comparisons in future research.

## 1. Introduction

Retrospective studies are inherently prone to case selection bias, which often undermines their scientific robustness. Despite this limitation, they remain valuable for rapidly collecting extensive data and addressing pressing clinical questions. The primary issue with retrospective studies lies in selection bias, which is a consequence of their design. Over time, patients with poor outcomes—such as those who succumbed to their conditions or were lost to follow-up—tend to be under-represented. This can skew the data, creating an overly optimistic depiction of patient outcomes and resulting in positively selected cohorts that fail to accurately reflect the broader reality. Another critical challenge in retrospective studies is the insufficient documentation of treatment-related toxicities. While hematological side effects such as neutropenia and thrombocytopenia are often documented due to their acute onset during therapy, non-hematological adverse events frequently go unrecorded in medical charts. This lack of comprehensive reporting further undermines the reliability of retrospective studies. As a result of these limitations, even retrospective studies with large sample sizes are often considered methodologically weaker and their findings undervalued. Nonetheless, retrospective studies are sometimes used as proof-of-concept frameworks to pave the way for prospective investigations, effectively serving as a validation tool for their own findings. This approach is particularly prevalent in registry studies conducted by prominent organizations such as European Society for Blood and Marrow Transplantation (EBMT) and Center for International Blood and Marrow Transplant Research (CIBMTR) [1,2,3]. However, many retrospective studies emerge directly from real-world clinical practice data collections. These studies are often standalone endeavors that are not followed by corresponding prospective studies, reflecting their pragmatic origins rather than adherence to traditional scientific inquiry.

Liposomal non-polymerized anthracycline (Myocet) has become a widely used treatment option in Italian hematological practices, facilitated by the provisions of Italian law 648/96. Italian law 648/96 authorizes the off-label use of medications for indications with demonstrated efficacy supported by clinical studies. In this context, non-pegylated liposomal anthracyclines are approved for patients receiving the R-CHOP regimen, particularly those aged over 65 or with pre-existing cardiac conditions that make the use of conventional anthracyclines inadvisable. Myocet is particularly beneficial for patients with pre-existing cardiac conditions or those over 65 years of age due to its reduced cardiotoxicity profile compared to standard anthracyclines, as demonstrated in oncological studies involving patients with breast cancer [4,5]. Anthracyclines, as an integral part of the CHOP regimen, have been pivotal in treating lymphoproliferative disorders since their introduction in the late 1970s [6], significantly contributing to the cure of various lymphoma types. However, their use is accompanied by substantial cardiotoxicity risks, notably congestive heart failure (CHF), which can manifest acutely during treatment or years later due to myocardial toxicity, leading to left ventricular dysfunction and a reduction in left ventricular ejection fraction [7,8,9]. The toxicity of anthracyclines is dose-dependent, with a cumulative doxorubicin dose of 550 mg/m^2^ being associated with a 30% risk of cardiovascular disease. Importantly, CHF and other cardiotoxic effects can occur even at lower cumulative doses of approximately 200 mg/m^2^.

The use of liposomal non-pegylated anthracyclines, originally employed in breast cancer treatment, has been shown to reduce cardiotoxicity in clinical studies [10]. This formulation was subsequently introduced in the treatment of aggressive lymphoma, replacing hydroxydaunorubicin in the CHOP schema (COMP regimen). Studies in hematology settings have confirmed the efficacy and safety of the COMP regimen in high-risk, negatively selected populations [11,12]. In a previous large-scale retrospective study conducted on behalf of the Fondazione Italiana Linfomi (FIL), we reported on the outcomes of R-COMP therapy in a highly selected cohort of patients [13]. The study showed comparable efficacy between R-COMP and historical data from R-CHOP, and demonstrated the feasibility of using anthracycline-based regimens in patients with pre-existing cardiac conditions, who would likely have been excluded from traditional treatments. Despite the inclusion of nearly 1000 patients, the retrospective nature of the study introduced limitations in the interpretation of its findings. Following this, FIL initiated a prospective study called the Elderly Project, aimed at collecting data on patients over 65 years of age with diffuse large B-cell lymphoma (DLBCL) from multiple Italian hematology centers. Over a span of 5 years, the project gathered data on more than 1000 patients, resulting in the publication of numerous papers [14], including one that evaluated the outcomes of patients treated with either R-CHOP or R-COMP [15]. The cohort of approximately 300 patients who received R-COMP is particularly noteworthy, as it consists of a consecutive and prospective group of individuals chosen for treatment with this regimen based on clinical criteria. These patients are representative of those in Italy eligible for non-pegylated liposomal anthracycline within the R-COMP regimen, as outlined by the provisions of Italian law 648/96.

Using the prospective cohort as a reference point allows us to better understand the extent of selection bias that may have affected the original retrospective study, thus improving the credibility and reliability of its findings. In this context, we applied a statistical hypothesis test to assess whether the patient group treated with non-pegylated liposomal anthracycline in the retrospective study [13] significantly differed from the cohort in the prospective study [15]. From a statistical perspective, our goal was to determine if the samples in both studies were drawn from the same underlying population. To address this, we employed a non-parametric statistical test known as the *k*-sample Anderson–Darling test. This test is designed to evaluate whether multiple random samples, which may vary in size, could have originated from the same unspecified distribution [16]. The statistic is computed under the assumption that the distribution function of each sample is continuous. In our analysis, we focused on “overall survival” as the continuous variable of interest for both the retrospective and prospective cohorts. We conducted this statistical test using the statistical software R (v. 4.4.1) [17] with the “kSamples” R package [18]. Specifically, we utilized the functions “ad.test” and “ad.test.combined” to perform the combined *k*-sample Anderson–Darling test and its combined version [16]. The combined version incorporated stratification based on clinically relevant variables, such as lymphoma stage and the International Prognostic Index (IPI), to minimize the influence of potential confounding factors. These variables were treated as blocks within the test framework, aligning with the principles of randomized block design.

## 2. Materials and Methods

To validate the retrospective data collection, we analyzed and compared two datasets. The first dataset originated from a retrospective study on the use of liposomal non-pegylated anthracycline in high-risk patients with diffuse large B-cell lymphoma (DLBCL) in Italy, who were treated with the R-COMP regimen under the provisions of Italian law 648/96. The findings of this study were published in Hematological Oncology in 2020 [13]. The second dataset was drawn from a prospective study, the Elderly Project (EP), which enrolled patients aged 65 and older with DLBCL, treating them with the same R-COMP regimen [15]. The retrospective dataset comprised 946 patients, while the prospective dataset included 308. It is important to highlight that the inclusion criteria for the two cohorts differed. The retrospective study encompassed all consecutive patients treated with R-COMP under the framework of Italian law 648/96, whereas the EP exclusively enrolled eligible patients aged 65 and older with DLBCL, from which we selected the R-COMP-treated cohort for analysis. As a result, the two groups differ significantly in terms of clinical and demographic characteristics and are not directly comparable.

Table 1 provides a summary of the demographic and clinical variables collected for each patient in the retrospective study. For each variable, the table includes a description and the corresponding coding scheme used for data analysis. Notably, the prospective study captured all the same variables as the retrospective study, with the exception of two: the therapy end date (DATA_FINETP) and the date of documented treatment response (DATA_RISPTP). Additionally, Table 2 presents the frequency distribution of the main variables in both the retrospective and prospective studies. Missing data are represented as NA. The variable sex exhibits a significant proportion of missing values, stemming from the design of the original study, where this information was deemed non-essential and recorded for only a limited subset of patients. Consequently, the variable sex was excluded from the statistical analysis in this study, ensuring that its absence does not influence the results presented here.

Our statistical analysis aimed to evaluate potential selection bias in the retrospective patient group treated with non-pegylated liposomal anthracycline by comparing it to prospectively enrolled patients. The primary objective was to determine whether the samples in both studies originated from the same underlying population. To address this, we employed a statistical hypothesis test. In 1987, Fritz Scholz and Michael A. Stephens introduced a method based on the Anderson–Darling measure of agreement between distributions, designed to assess whether multiple random samples, possibly of differing sizes, may have been drawn from the same unspecified distribution [16]. In this work, we applied the *k*-samples Anderson–Darling test and the combined version of the *k*-samples Anderson–Darling test to compare the two studies. The application was performed in the statistical software R (v. 4.4.1) [17] using the R package “kSamples” [18]. Specifically, we employed the functions “ad.test” and “ad.test.combined” to perform, respectively, the combined *k*-sample Anderson–Darling test and its combined version [16]. The results are discuss in the Section 3 of this work.

The *k*-samples Anderson–Darling test is a rank test. The underlying assumptions are as follows: (i) all observations are independent in the *k*-samples and (ii) each sample has continuous distribution function $F_{i}$, i=1,…,k. Moreover, we denote the number of observations of the pooled sample with *N*, $N=n_{1}+\cdots +n_{k}$, and $Z_{1}<Z_{2}<\cdots <Z_{N}$ the pooled ordered sample. The null hypothesis is that all samples have the same common distribution *F*:

$$H_{0}:F=F_{1}=\cdots =F_{k}$$

and the rank statistics is defined as

$$A_{kN}^{2}=\frac{1}{N}\sum_{i=1}^{k}\sum_{j=1}^{N-1}\frac{{\left(NM_{ij}-jn_{i}\right)}^{2}}{j(N-j)}$$

where $M_{ij}$ is the number of observations in the *i*-th sample that is not greater than $Z_{j}$.

In our analysis, we examined two distinct samples (*k* = 2): the prospective cohort and the retrospective study’s patients. The first assumption of the Anderson–Darling test, independence of the samples, is satisfied, as the two studies are unrelated. The second assumption is also fulfilled, as we selected overall survival as a continuous variable to compare the distributions between the two cohorts. Overall survival was calculated by subtracting the diagnosis date from the follow-up date, with the result expressed in months. To address age as a potential confounding factor in survival analysis, the patient cohort in the retrospective study was restricted to individuals aged 65 years and older. This adjustment resulted in a dataset comprising 757 cases from the retrospective study and 308 cases from the prospective study. No missing data were identified for diagnosis or follow-up dates in either study. As a result, the calculated overall survival variable is complete and contains no missing values.

Table 3 provides the frequency distribution of key variables in both cohorts, specifically focusing on patients aged 65 years or older. Missing data are reported as NA. Independent *k*-sample Anderson–Darling tests can be combined, allowing *k* to vary between groups of samples and permitting the common distribution function to differ across groups. The combined version of the *k*-sample Anderson–Darling test is particularly useful in contexts such as analyzing treatment effects in randomized block experiments or assessing performance equivalence across multiple laboratories when presented with diverse test materials [18]. In this work, we applied the combined *k*-sample Anderson–Darling test to mitigate potential systematic errors, such as variations between different subsets of data. To adapt this approach to our scenario, we treated the two studies as separate “laboratories” and utilized the variables *stage of lymphoma* and IPI as factor variables to define different blocks or *levels*. By selecting *stage of lymphoma* and *IPI* as level variables, we ensured that our analysis adhered to the assumptions of randomized block experiments, particularly the random assignment of treatments within each group. Notably, *stage of lymphoma* and *IPI* can be considered randomly assigned to patients, satisfying this assumption. These categorical variables were chosen because they represent intrinsic clinical characteristics that are unaffected by a physician’s intervention, thereby providing an unbiased framework for our analysis. As reported in Table 3, the *stage of lymphoma* exhibited a low rate of missing data (0.4%) in the retrospective study. In contrast, the *IPI* score demonstrated a moderate level of missing information, with 3% and 7% missing values observed in the retrospective and prospective studies, respectively.

Finally, we performed the classic Pearson’s $\chi^{2}$ test to compare the distribution of the categorical variables *IPI* and *stage of lymphoma* between the two studies.

## 3. Results

Table 4 presents the results of applying the R function “ad.test” to perform the Anderson–Darling *k*-sample test on our data, which enables us to compare the overall survival distribution in both studies. Based on the *p*-values obtained from both versions of the test statistic, we reject the null hypothesis of equal distribution.

We applied the combined version of the *k*-sample Anderson–Darling test to evaluate the overall survival distributions of patients grouped by *stage of lymphoma* and IPI scores in the two studies. In Table 5 and Table 6, which display the outputs of the R function “ad.test.combined”, “Dataset 1” refers to the prospective study, while “Dataset 2” represents the retrospective study. Table 5 summarizes the test results for patients stratified by lymphoma stage. The *p*-values for both test statistics indicate that the null hypothesis of equal distributions cannot be rejected, either within each dataset or between the two studies. This finding suggests that the overall survival distributions by lymphoma stage are consistent both within and across the two cohorts. In contrast, the results for IPI scores, shown in Table 6, reveal a different outcome. Here, the *p*-values from both test statistics indicate that the null hypothesis of equal distributions can be rejected, both within each dataset and between the two studies. These results suggest significant differences in the overall survival distributions when stratified by IPI score, both across and within the cohorts.

Finally, the results of the classic Pearson’s $\chi^{2}$ test, used to compare the distributions of the categorical variables *IPI* and *stage of lymphoma* between the two studies, largely support the findings from the combined *k*-sample Anderson–Darling tests. Specifically, the *p*-value (0.1072) from the Pearson’s $\chi^{2}$ test, assessing the distribution of lymphoma stage in the two studies, indicates no statistically significant difference between the distributions. In contrast, the Pearson’s $\chi^{2}$ test (significance level α=0.05) for IPI scores revealed a statistically significant difference (*p*-value = 0.03153) between the two studies. The results from two of the three statistical tests that were conducted (Table 4, Table 5 and Table 6) lead to the rejection of the null hypothesis of homogeneity. This finding suggests the possibility of unintentional selection bias in the retrospective study, where patients may have been inadvertently selected based on uncontrolled factors. The results of the combined *k*-sample Anderson–Darling test, using the IPI score as a level variable, and the Pearson’s $\chi^{2}$ test, which compares IPI score distributions between the two studies, may be affected by missing data in the factor variable. It is important to note that these statistical tests automatically exclude observations with missing data. As a result, the rejection of the null hypothesis in both tests is based on the available data distribution, rather than the complete one.

Our data partially support this concern, as there is a notable difference in the distribution of IPI scores between the two studies. Specifically, the retrospective study has fewer high-risk IPI scores compared to the prospective study (Table 2). On the other hand, the combined *k*-sample Anderson–Darling test using the stage of lymphoma as a level variable (Table 5) does not reject the null hypothesis. This result implies that the two groups (retrospective and prospective) may come from the same population, which would suggest no selection bias in this aspect of the analysis.

## 4. Discussion

Retrospective studies have long been a cornerstone in medical research, providing invaluable insights, especially when large patient cohorts are involved. These studies serve as a foundation for the development of prospective studies, often allowing researchers to generate hypotheses and establish trends. However, the use of retrospective studies is not without limitations, including the potential for selection biases. Although large sample sizes can mitigate the impact of such biases, it remains essential to understand these limitations when interpreting results [19]. In the near future, artificial intelligence (AI)-generated synthetic patient cohorts may become an alternative to traditional retrospective studies, further emphasizing the need for a robust understanding of both retrospective and prospective study methodologies [20]. This study was designed to apply statistical analysis to assess whether large retrospective case studies can serve as a reliable representation of the general population, with a particular focus on addressing selection bias.

The primary objective of this study was to validate a large retrospective study published in 2020 [13] by comparing its findings with a more recent prospective study, the Elderly Project (EP) [15]. The retrospective study aimed to demonstrate the reduced risk of cardiotoxicity associated with the use of non-pegylated liposomal anthracycline in non-Hodgkin lymphomas. Validating the retrospective study’s findings is crucial, as they may serve as a valuable reference for future comparative research. While retrospective studies offer important contributions to the scientific community, they are often vulnerable to selection bias, which is frequently unintentional and goes unnoticed.

Our analysis partially confirms this concern, as we observed a lower proportion of high-risk IPI scores in the retrospective cohort compared to the prospective cohort. This suggests a possible discrepancy in patient selection between the two cohorts, a finding reinforced by some of our statistical analyses. However, the application of the combined version of the *k*-sample Anderson–Darling test suggests that the two groups (retrospective and prospective) may originate from the same underlying population. This discrepancy does not necessarily indicate bias in the retrospective cohort, but it may reflect limitations in the statistical approach, as well as in the quality of data used in our analysis. Several factors contribute to these discrepancies: (i) the assumption of a continuous distribution led to the selection of overall survival as the comparison variable, despite the fact that this variable is influenced by various factors and is not entirely objective [21]; (ii) the statistical tests employed may have lacked sufficient power to detect meaningful differences; and (iii) the results were susceptible due to missing data. A more comprehensive understanding of the true data distribution, including observations with missing values, could potentially yield different statistical outcomes. This issue is particularly relevant in cases where the *p*-value is close to the significance threshold, as observed in Pearson’s $\chi^{2}$ test for the IPI variable in Table 3 and Dataset 1 in Table 6.

Our findings point to the importance of addressing the limitations associated with missing data in retrospective studies. Future work could explore alternative statistical hypothesis tests for comparing the distributions of categorical variables, potentially improving the reliability of such comparisons. Additionally, the methodology developed in this study could be extended to facilitate comparisons between other large retrospective case studies and prospective cohorts. Such analyses would contribute to a broader comparative evaluation of results across different studies, offering valuable insights into methodological consistency, data interpretation, and the generalizability of findings. By exploring these avenues, we can enhance the precision and applicability of research findings in clinical settings [22].

### Recommendations for Future Research

To further improve the reliability and robustness of comparisons between retrospective and prospective cohorts, future studies should consider the following:

- Alternative statistical methods: Explore the use of alternative statistical tests that may be better suited to handling non-continuous or skewed data distributions, such as non-parametric methods or machine learning approaches for data imputation and comparison.
- Incorporation of artificial intelligence (AI): AI-driven techniques could be employed to analyze large datasets, helping to identify and mitigate biases, particularly in cases of missing data or unmeasured confounders. These methods could also generate synthetic cohorts to supplement traditional retrospective studies.
- Longitudinal studies and data quality: Future studies should aim for improved data quality, particularly in terms of completeness and consistency of variables, such as clinical outcomes and patient demographics. Longitudinal designs may also help to track outcomes over extended periods, providing more detailed insights into long-term effects.
- Multicenter and international studies: Expanding the scope of studies to include multicenter and international collaborations could enhance the generalizability of findings and provide a broader perspective on treatment efficacy across diverse patient populations.

## 5. Conclusions

This study contributes to the growing body of research examining the validity of retrospective studies, particularly regarding their comparison to prospective cohorts. By applying statistical methods to assess potential selection bias, this research provides valuable insights into the reliability of retrospective case studies as representations of the general population. The findings underscore both the strengths and limitations of the current statistical tools, highlighting the need for continued development of more precise methods. Ultimately, this work serves as a starting point for future research focused on refining these techniques and improving the robustness of retrospective studies, ensuring that they provide reliable data that can be confidently used to inform clinical decisions and future studies.

## Figures and Tables

**Table 1:** Description of the variables collected in the retrospective study.

Variable	Description	Format
CENTRO	Health center and data collection site	String
DATA_NASC	Date of birth of the patient	Date: “yyyy-mm-dd”
SESSO	Gender of the patient	Binary: 1 = male 2 = female
ETA	Patient’s age at the time of diagnosis (in years)	Numeric
DATA_DIAG	Diagnosis date	Date: “yyyy-mm-dd”
STADIO	Stage of the lymphoma	Factorial: from 1 to 4
SINTOMI	Presence/absence of systemic symptoms	Binary: 1 = No symptoms 2 = symptoms
IPI	International Prognostic Index	Factorial: From 0 to 3
MOT_USOGOMP	Reason to use R-COMP	Factorial: 1 = Age 2 = Cardiac disease 3 = Previous use of anthracycline 4 = Isotype 5 = Controlled hypertension without stroke 6 = Severe arrhythmias
DATA_FINETP	End of therapy date	Date: “yyyy-mm-dd”
RISPOSTA_TP	Response to therapy (R-COMP)	Factorial: 0 = Complete Remission, 1 = Partial Remission, 2 = Non-response/progression, 3 = Non-evaluable for sudden death.
DATA_RISPTP	Date of treatment response (R-COMP) was documented	Date: “yyyy-mm-dd”
STATO	Health status at follow-up date	Factorial: 0 = Alive 1 = Dead 2 = Lost to follow-up
RELAPSE	Relapse	Factorial: 0 = No, 1 = Yes, 2 = Never in Remission
DATA_REC/PROG	Relapse date for patients in complete remission (RISPOSTA_TP = 0) or progression date for patients in partial remission or non-responders (RISPOSTA_TP = 1 or 2)	Date: “yyyy-mm-dd”
DATA_FU	Follow-up date, which corresponds to the date of death for deceased patients	Date: “yyyy-mm-dd”
CAUSA_MORTE	Cause of death	Factorial: 0 = Alive 1 = Lymphoma 2 = Non-cardiac therapy complication 3 = Acute cardiac episode 4 = Unknown/Not specified 5 = New neoplasm
TRT2	Time, in months, from the diagnosis date to the follow-up date.	Date: “yyyy-mm-dd”

**Table 2:** Distribution of key variables across the retrospective and prospective studies, comparing patient characteristics, clinical features, and treatment outcomes between the two cohorts.

Variable	Retrospective *n* (*p*)	Prospective *n* (*p*)
Age in classes:		
0–65	120 (14%)	0 (0%)
65–69	131 (15%)	40 (13%)
70–79	478 (54%)	201 (65%)
80–89	143 (16%)	67 (22%)
90–95	5 (1%)	0 (0%)
NA	69 (7%)	0 (0%)
Sex:		
1 = male	284 (53%)	153 (50%)
2 = female	252 (47%)	155 (50%)
NA	410 (43%)	0 (0%)
Stage of lymphoma:		
1	115 (12%)	33 (11%)
2	189 (20%)	63 (20%)
3	203 (22%)	53 (17%)
4	435 (46%)	159 (52%)
NA	4 (0.5%)	0 (0%)
IPI:		
0	184 (20%)	60 (21%)
1	282 (31%)	69 (24%)
2	288 (31%)	88 (31%)
3	162 (18%)	70 (24%)
NA	30 (3%)	21 (7%)
Response to therapy:		
0 = Complete remission	687 (72%)	201 (66%)
1 = Partial remission	119 (13%)	52 (17%)
2 = Non-response/progression	134 (14%)	30 (10%)
3 = Non-evaluable for sudden death	6 (1%)	23 (7%)
NA	0 (0%)	2 (0.7%)
Presence/absence of systemic symptoms:		
1 = No symptoms	739 (81%)	216 (70%)
2 = Symptoms	174 (19%)	92 (30%)
NA	33 (4%)	0 (0%)
Relapse:		
0 = No	566 (60%)	206 (67%)
1 = Yes	129 (14%)	81 (26%)
2 = Never in Remission	251 (26%)	21 (7%)
NA	0 (0%)	0 (0%)
Cause of death:		
Alive	614 (65%)	231 (75%)
Lymphoma	207 (22%)	57 (19%)
Acute cardiac episode	16 (2%)	0 (0%)
Other causes	109 (11%)	20 (6%)
NA	0 (0%)	0 (0%)

**Table 3:** Frequency distribution of key variables in the retrospective and prospective studies, with the retrospective cohort being restricted to patients aged 65 or older.

Variable	Retrospective *n* (*p*)	Prospective *n* (*p*)
Age in classes:		
65–69	131 (17%)	40 (13%)
70–79	478 (63%)	201 (65%)
80–89	143 (19%)	67 (22%)
90–95	5 (1%)	0 (0%)
NA	0 (0%)	0 (0%)
Sex:		
1 = male	229 (52%)	153 (50%)
2 = female	214 (48%)	155 (50%)
NA	314 (42%)	0 (0%)
Stage of lymphoma:		
1	97 (13%)	33 (11%)
2	161 (21%)	63 (20%)
3	166 (22%)	53 (17%)
4	332 (44%)	159 (52%)
NA	1 (0.1%)	0 (0%)
IPI:		
0	145 (20%)	60 (21%)
1	233 (31%)	69 (24%)
2	232 (31%)	88 (31%)
3	131 (18%)	70 (24%)
NA	16 (2%)	21 (7%)
Response to therapy:		
0 = Complete remission	552 (73%)	201 (66%)
1 = Partial remission	89 (12%)	52 (17%)
2 = Non-response/progression	111 (14%)	30 (10%)
3 = Non-evaluable for sudden death	5 (1%)	23 (7%)
NA	0 (0%)	2 (0.7%)
Presence/absence of systemic symptoms:		
1 = No symptoms	589 (80%)	216 (70%)
2 = Symptoms	146 (20%)	92 (30%)
NA	22 (3%)	0 (0%)
Relapse:		
0 = No	452 (60%)	206 (67%)
1 = Yes	104 (14%)	81 (26%)
2 = Never in Remission	201 (26%)	21 (7%)
NA	0 (0%)	0 (0%)
Cause of death:		
Alive	485 (64%)	231 (75%)
Lymphoma	175 (23%)	57 (19%)
Acute cardiac episode	10 (1%)	0 (0%)
Other causes	87 (12%)	20 (6%)
NA	0 (0%)	0 (0%)

**Table 4:** *k*-sample Anderson–Darling test.

Number of samples: 2			
Sample sizes: 308, 757			
Number of ties: 221			
Mean of Anderson–Darling Criterion: 1			
Standard deviation of Anderson–Darling criterion: 0.76009			
T.AD = (Anderson–Darling criterion − mean)/sigma			
Null hypothesis: All samples come from a common population.			
	AD	T.AD	asympt. *p*-value
version 1:	22.138	27.810	2.1177 × 10^−12^
version 2:	22.200	27.831	2.2051 × 10^−12^

*Note*: *k*-sample Anderson–Darling test.

**Table 5:** Combination of two independent *k*-sample Anderson–Darling tests applied to the *stage of lymphoma* as a categorical variable.

Sample sizes within each dataset:			
Dataset 1: 33 63 53 159			
Dataset 2: 97 161 166 332			
Total sample size per dataset: 308 756			
Number of unique values per dataset: 279 642			
AD.i = Anderson–Darling criterion for i-th dataset			
Means: 3 3			
Standard deviations: 1.30713 1.31388			
T.i = (AD.i − mean.i)/sigma.i			
Null hypothesis: All samples within a dataset come from a common distribution.			
The common distribution may change between datasets.			
For Dataset 1, we obtain			
	AD	T.AD	asympt. *p*-value
version 1:	4.9182	1.4675	0.084444
version 2:	4.9300	1.4756	0.083727
For Dataset 2, we obtain			
	AD	T.AD	asympt. *p*-value
version 1:	3.7171	0.54579	0.23577
version 2:	3.7300	0.55548	0.23342
Combined Anderson–Darling criterion: AD.comb = AD.1 + AD.2			
Mean = 6 Standard deviation = 1.85334			
T.comb = (AD.comb − mean)/sigma			
	AD.comb	T.comb	asympt. *p*-value
version 1:	8.6353	1.4219	0.088954
version 2:	8.6600	1.4352	0.087501

*Note*: Combined *k*-sample Anderson–Darling tests.

**Table 6:** Combination of two independent *k*-sample Anderson–Darling tests. *IPI* as level variable.

Sample sizes within each dataset:			
Dataset 1: 60 69 88 70			
Dataset 2: 145 233 232 131			
Total sample size per dataset: 287 741			
Number of unique values per dataset: 262 635			
AD.i = Anderson–Darling criterion for i-th dataset			
Mean: 3 3			
Standard deviations: 1.30547 and 1.31365			
T.i = (AD.i − mean.i)/sigma.i			
Null hypothesis: All samples within a dataset come from a common distribution.			
The common distribution may change between datasets.			
For Dataset 1, we obtain			
	AD	T.AD	asympt. *p*-value
version 1:	6.6066	2.7627	0.018224
version 2:	6.6400	2.7860	0.017743
For Dataset 2, we obtain			
	AD	T.AD	asympt. *p*-value
version 1:	16.962	10.629	4.8262 × 10^−7^
version 2:	17.000	10.635	4.7151 × 10^−7^
Combined Anderson–Darling criterion: AD.comb = AD.1 + AD.2			
Mean = 6 Standard deviation = 1.85201			
T.comb = (AD.comb − mean)/sigma			
	AD.comb	T.comb	asympt. *p*-value
version 1:	23.569	9.4863	2 × 10^−7^
version 2:	23.640	9.5248	2 × 10^−7^

*Note*: Combined *k*-sample Anderson–Darling tests.

## Data Availability

The data presented in this study are available on request from the corresponding author. The data are not publicly available due to privacy and ethical restrictions.
